# Prognostic factors of malignant peritoneal mesothelioma: a retrospective study of 52 female patients

**DOI:** 10.1186/s12957-022-02688-x

**Published:** 2022-06-29

**Authors:** Jianting Ma, Shengzhi Zhang

**Affiliations:** Obstetrics and Gynecology Department, Yuyao People’s Hospital of Zhejiang Province, Yuyao, 315400 Zhejiang China

**Keywords:** Cytoreductive surgery, Female malignant peritoneal mesothelioma, Pathological subtype, Pemetrexed, Prognosis

## Abstract

**Background:**

Prognosis in malignant peritoneal mesothelioma (MPM) remains poor, and the associated factors are unclear. Therefore, this study aimed to investigate the prognostic factors of MPM.

**Methods:**

A total of 52 female MPM patients treated in 2012–2017 were retrospectively analyzed. Kaplan-Meier survival curves were generated for survival analysis by the log-rank test. The Cox regression model was used for univariate and multivariate analyses.

**Results:**

Univariate analysis showed that median survival time (MST) was longer in the epithelioid type compared with the sarcomatoid type (12 months vs 5 months); cumulative survival rates at 12 months were 45.7% and 0%, respectively (*P=*0.005). MST was longer in patients with proliferating cell nuclear antigen (Ki67) ≤ 10% compared with those with Ki67 > 10% (15 months vs 11 months). Cumulative survival rates at 12 months were 60.0% and 28.1%, respectively (*P=*0.036). MSTs in patients administered peritoneal biopsy or adnexectomy + paclitaxel + platinum perfusion, peritoneal biopsy (or adnexectomy) + pemetrexed + platinum perfusion, cytoreductive surgery + paclitaxel + platinum perfusion, and cytoreductive surgery + pemetrexed + platinum perfusion were 6, 11, 12, and 24 months, respectively, with cumulative survival rates at 12 months of 0%, 35.7%, 45.5%, and 73.3%, respectively. Survival time after cytoreductive surgery combined with pemetrexed + platinum was the longest. In multivariate analysis, pathological type, T staging, and therapeutic regimen were independent prognostic factors of MPM (*P <* 0.05).

**Conclusions:**

Prognosis in MPM is associated with pathological subtype, clinical staging, cytoreductive surgery, and subsequent pemetrexed use. Radical cytoreductive surgery and postoperative use of pemetrexed prolong survival.

## Background

Mesothelioma represents an extremely rare and highly malignant tumor affecting serosal membranes such as the pleura, peritoneum, pericardium, and the tunica vaginalis of the testes [1]. Malignant mesothelioma is scarce, with most cases arising from the pleura and malignant peritoneal mesothelioma (MPM) comprising 7–30% of all cases [2]. The World Health Organization classifies this disease into epithelioid, sarcomatoid, and mixed types [3, 4]. Epidemiological evidence shows that the annual incidence of MPM is 1–2 per 1 million individuals [5]; meanwhile, in the Czech population, the age-adjusted incidence of primary peritoneal tumors was 4.36/year/1,000,000 inhabitants between 2012 and 2016, versus 99.0/year/1,000,000 inhabitants for synchronous secondary peritoneal cancers in 2014–2016 [6]. It is known that women in Yuyao show an incidence of approximately 5.7/1 million [7]. This apparent female predominance can be explained by that many handicraft workshops producing asbestos were established in this area in the 1970s and 1980s, mostly employing women.

There are no known specific clinical symptoms and signs for MPM at the early disease stage, which explains why it is usually diagnosed at an advanced stage [8]. However, a systematic review revealed nucleoplasmin 2 (NPM2) is associated with MPM, indicating a critical role for NPM2 in the development and progression of MPM [9], which could be used for early diagnosis. The prognosis of MPM patients is extremely poor. Indeed, abdominal complications due to systemic metastasis or intestinal dystrophy cause death in many cases; left untreated, the life expectancy of patients is less than 1 year, and there is currently no consensus on the treatment of this malignancy, although multiple studies have utilized intraperitoneal hyperthermic chemotherapy (HIPE) in combination with intravenous chemotherapy or cytoreductive surgery (CRS) [10–14]. Therefore, prolonging survival time in MPM patients and identifying the associated prognostic factors are difficult problems for researchers.

Given the current shortage in identifying factors associated with MPM, and the abnormal prevalence rate in Yuyao city, the present study aimed to explore the prognostic factors of MPM in females and the clinical therapy that could prolong survival the most.

## Methods

### Patients

A total of 52 female MPM patients treated in our hospital from January 2012 to December 2017 were retrospectively assessed. Inclusion criteria were (1) 18- to 75-year-old female; (2) clinical diagnosis determined by pathology, in accordance with the 2012 US “Mesothelioma Pathology Diagnostic Guidelines” [15] and “Peritoneal mesothelioma: PSOGI/EURACAN clinical practice guidelines for diagnosis, treatment and follow-up” [16]; (3) no serious complications, including severe heart disease, liver disease, and renal insufficiency; and (4) treatment by tumor reduction surgery or conventional chemotherapy. Patients with incomplete data were excluded. This study was approved by the Medical Ethics Committee of our Hospital (approval number: 2021-08-001; date: August 19, 2021). Informed consent was waived due to the retrospective design. All procedures were performed in accordance with the ethical standards laid down in the 1964 Declaration of Helsinki and its later amendments.

### Clinical measurements

All patients were evaluated by laparoscopy or laparotomy to detect pathological subtypes. Pathological diagnosis was performed by two deputy chief physicians with 21 and 30 years of experience in pathology, respectively. According to the peritoneal cancer index (PCI) score proposed by Jacquet et al. in 1996 [17], intraoperative tumor burden was evaluated. The abdomen was divided into 13 regions, and scored as follows: 0 point, no macroscopic tumor; 1 point, tumor diameter ≤ 0.5 cm; 2 points, tumor diameter of 0.5–5.0 cm; 3 points, tumor diameter > 5.0 cm or tumor fusion. The sum of the above scores was considered the PCI (0–39 points). T staging was based on a multi-center clinical analysis of MPM [18].

Postoperative specimens were fixed with 10% neutral formalin, paraffin-embedded, and sectioned. Immune cells were detected by the SP method (Fuzhou Maixin Biotechnologies Development Company, China). Two senior doctors performed double-blind film reading, and immunostaining was performed. Then, 3 high-power fields were randomly selected in each section, and the average proportion of positive cells was obtained: < 5%, negative; 5–25%, “+”; 26–50%, ++; >50%, “+++.” Ki67 ≤ 10% and > 10% were considered to be low and high, respectively. According to reports by Pezzuto F et al. [19, 20], Pillai K et al. [21], and Kusamura S et al. [22], and the *Chinese Expert Consensus on the Diagnosis and Treatment of Diffuse Malignant Peritoneal Mesothelioma*, Ki67 >9% is a relative contraindication for cytoreductive surgery (CRS) + hyperthermic intraperitoneal chemotherapy (HIPEC). Therefore, Ki67=10% was considered the cutoff value in this study.

### Treatment

The patients were treated immediately after a definite diagnosis. According to the treatment plan, the patients were divided into the tumor reduction surgery and non-tumor reduction surgery groups, administered tumor reduction therapy combined with chemotherapy and conventional chemotherapy alone, respectively. All surgical patients were operated by surgeons with more than 21 years of surgical experience. As a postoperative chemotherapy regimen, the paclitaxel + carboplatin regimen was used in one ward, versus the pemetrexed + carboplatin regimen in the other. Therefore, there were four treatment options: (G1) peritoneal biopsy (or adnexectomy) combined with paclitaxel + platinum perfusion; (G2) peritoneal biopsy (or adnexectomy) combined with pemetrexed + platinum perfusion; (G3) cytoreductive surgery combined with paclitaxel + platinum perfusion; and (G4) cytoreductive surgery combined with pemetrexed + platinum perfusion.

The range of cytoreductive surgery included the whole uterus, bilateral adnexa, the greater omentum, and the lesser omentum, with anterior and posterior pelvic peritoneum resection, as well as small mesenteric tumor resection. The residual gross tumor was < 0.5-1 cm. The Sugarbaker completeness of cytoreduction (CCR) scoring method was used to evaluate intraoperative CCR [17]. The above chemotherapy cycles were all performed for 21 days, with 3 to 9 cycles; in patients with less than 6 cycles, treatment was discontinued due to disease progression. The doses of chemotherapy drugs were: paclitaxel, 175 mg/m^2^; cisplatin, 80 mg/m^2^, or an area under the curve for carboplatin of 5; pemetrexed, 500 mg/m^2^.

### Outcomes and follow-up

The effects of the four treatment options were evaluated by the median survival time (MST), starting from the time of diagnosis to death or last follow-up on December 31, 2019. During the chemotherapy cycle, follow-up was performed on average at 21-day intervals, and 2 months after chemotherapy completion. Follow-up exams included full-abdomen enhanced CT and the assessment of related serological indicators.

### Statistical methods

The SPSS13.0 software (SPSS, Chicago, IL, USA) was used for data analysis. Measurement data with normal distribution are mean ± SD and were compared by the *t*-test. Non-normally distributed measurement data were represented by median (range) and compared by the non-parametric Mann-Whitney *U* test. Count data were displayed as number and percentage and assessed by the chi-square test. Variables with *P* < 0.05 in the single-factor COX model were included in the multi-factor COX regression model and filtered by the Backward selection method.

## Results

### Patient baseline characteristics

A total of 52 patients were included in this study, with an average age of 60.63 ± 10.32 years (ranging from 42 to 75 years). There were 46 and 6 patients with epithelioid and sarcomatoid types, respectively. A total of 44 patients had a previous history of asbestos exposure for different durations (1–12 years), while 8 had no asbestos exposure history. All baseline patient data are summarized in Table 1.

**Table 1:** Baseline patient features

Variables	All patients (*n*=52)
Age, years	60.63 ± 10.32
BMI, kg/m^2^	24.56 ± 8.42
History of asbestos exposure	
No	8
Yes	44
Treatment programs	
G1	12
G2	14
G3	11
G4	15
T staging	
T3	36
T4	16
Pathological type	
Epithelioid	46
Sarcomatoid	6
Ki67	
≤10%	20
>10%	32
PCI score	
0–10	0
11–20	0
21–30	36
31–39	16

*G1* peritoneal biopsy (or adnexectomy) combined with paclitaxel + platinum perfusion, *G2* peritoneal biopsy (or adnexectomy) combined with pemetrexed + platinum perfusion, *G3* cytoreductive surgery combined with paclitaxel + platinum perfusion, *G4* cytoreductive surgery combined with pemetrexed + platinum perfusion

### Analysis of overall survival of female MPM patients

The median survival time of the 52 patients was 12.0 months (95% CI: 9.7–14.3); there were 50 deaths (96.2%), 2 patients surviving with tumors (3.8%), and no loss to follow-up.

### Univariate analysis of prognostic factors in MPM patients

Univariate analysis results of pathological type, Ki67, therapeutic regimen, and prognosis of MPM patients are shown in Table 2.

**Table 2:** Univariate analysis of pathological type, Ki67, therapeutic regimen, and prognosis of MPM patients

Variables	Median survival time (month), 95% CI	*P* value
All patients	12 (9.7~14.3)	
History of asbestos exposure		
No	12 (4.6~19.4)	0.537
Yes	11 (7.8~14.3)	
T staging		
T3	12 (9.5~14.5)	0.023
T4	6 (4.7~7.3)	
Pathological type		0.005
Epithelioid	12 (10.1~13.9)	
Sarcomatoid	5 (2.0~9.8)	
Ki67		0.036
≤10%	15 (12.1~17.9)	
>10%	11 (7.1~14.9)	
Therapeutic regimen		<0.001
G1	6 (3.8~8.2)	
G2	11 (3.7~18.3)	
G3	12 (8.0~16.0)	
G4	24 (20.0~28.0)	

*G1* peritoneal biopsy (or adnexectomy) combined with paclitaxel + platinum perfusion, *G2* peritoneal biopsy (or adnexectomy) combined with pemetrexed + platinum perfusion, *G3* cytoreductive surgery combined with paclitaxel + platinum perfusion, *G4* cytoreductive surgery combined with pemetrexed + platinum perfusion

### Survival times in patients administered different therapeutic regimens

**Fig. 1:**
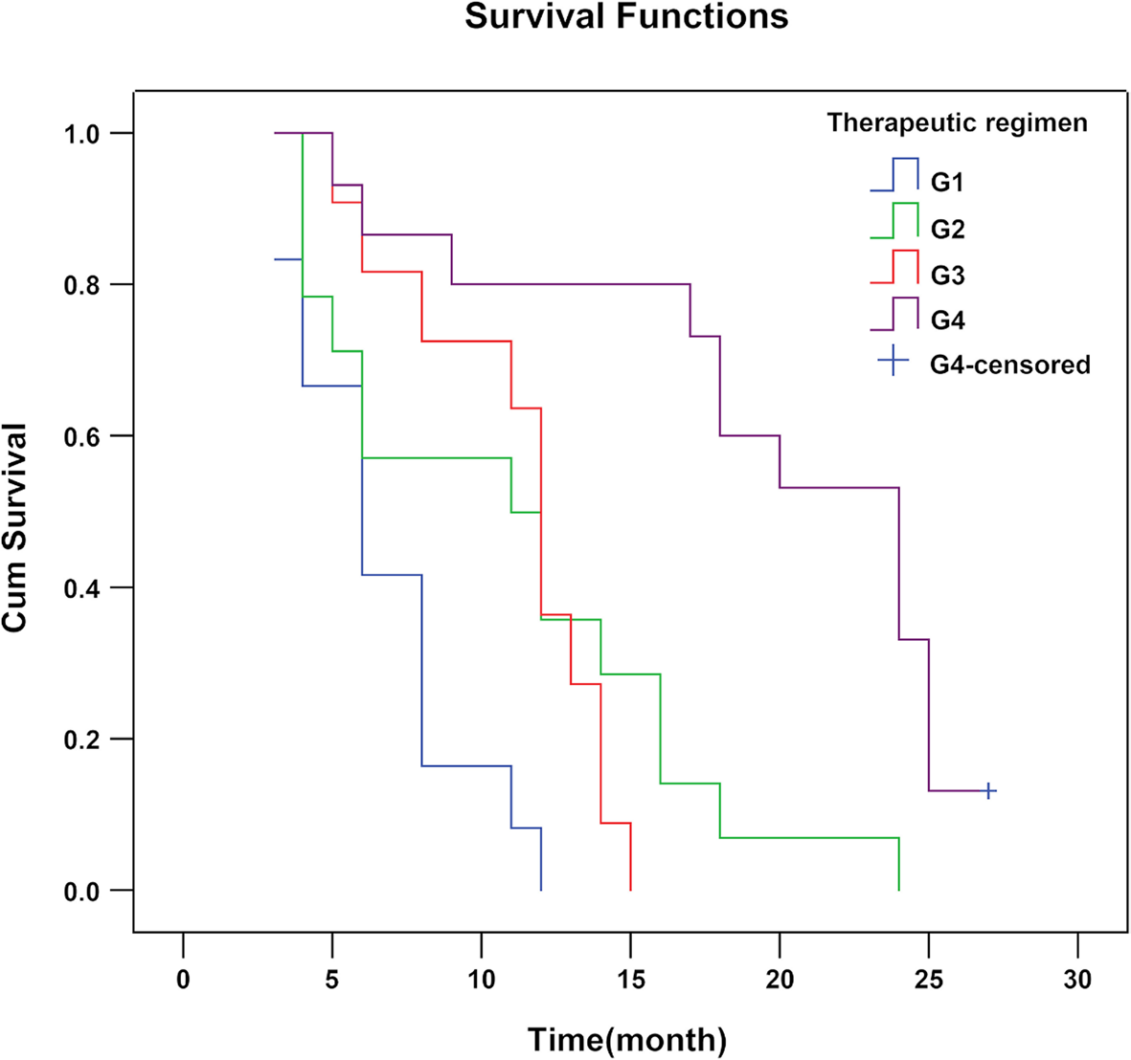
Survival curve analysis of different therapeutic regimens. G1, peritoneal biopsy (or adnexectomy) combined with paclitaxel + platinum perfusion; G2, peritoneal biopsy (or adnexectomy) combined with pemetrexed + platinum perfusion; G3, cytoreductive surgery combined with paclitaxel + platinum perfusion; G4, cytoreductive surgery combined with pemetrexed + platinum perfusion

MSTs in patients administered peritoneal biopsy or adnexectomy + paclitaxel + platinum perfusion, peritoneal biopsy (or adnexectomy) + pemetrexed + platinum perfusion, cytoreductive surgery + paclitaxel + platinum perfusion, and cytoreductive surgery + pemetrexed + platinum perfusion were 6, 11, 12, and 24 months, respectively, with cumulative survival rates at 12 months of 0%, 35.7%, 45.5%, and 73.3%, respectively. The differences among the four groups were statistically significant (*χ*^2^ = 30.00, *P* < 0.001, Fig. 1). The survival time of patients administered cytoreductive surgery combined with pemetrexed + platinum was the longest. MST and the cumulative survival rate at 12 months were increased in patients administered cytoreductive surgery combined with paclitaxel + platinum compared with the peritoneal biopsy (or adnexectomy) + pemetrexed + platinum group, although the difference was not statistically significant (*χ*^2^ = 0.09, *P* = 0.765).

### Pathological type and patient survival

After pathological diagnosis, according to WHO classification criteria, there were 46 with epithelioid MPM, and 6 with the sarcomatoid type. In the epithelioid group, MST was 12 months, with a cumulative survival rate at 12 months of 45.7%. In the sarcomatoid type, MST was 5 months, and the cumulative survival rate at 12 months was 0%, indicating statistically significant differences between the two groups (*P* = 0.005).

### Expression levels of Ki67 and patient survival

Specimens underwent pathological diagnosis and immunohistochemical staining, with double-blind film reading. MSTs in patients with Ki67 ≤ 10% and Ki67 > 10% were 15 and 11 months, respectively; the cumulative survival rates at 12 months were 60.0% and 28.1%, respectively. These findings indicated that patients with Ki67 ≤ 10% had longer MST and higher cumulative survival rate at 12 months compared with the Ki67 > 10% group (*χ*^2^ = 4.374, *P* = 0.036).

### Multivariate analysis of MPM patient prognosis

T staging, Ki67, pathological type, and therapeutic regimen were selected as independent variables and survival time as a dependent variable. Then, the Cox risk proportional function model was used to conduct a multivariate analysis of potential prognostic factors of MPM (Table 3).

**Table 3:** Potential prognostic factors of MPM

Prognostic factors	Hazard ratio, 95% CI	*P* value
Ki67		
>10%	Ref	
≤10%	0.945 (0.489~1.826)	0.867
T staging		
T3	Ref	
T4	2.024 (1.032~3.971)	0.040
Pathological type		
Epithelioid	Ref	
Sarcomatoid	7.663 (2.715~21.625)	<0.001
Therapeutic regimen		
G4	Ref	
G1	22.794 (7.302~71.149)	<0.001
G2	5.797 (2.312~14.538)	<0.001
G3	4.823 (1.735~13.405)	0.003

*G1* peritoneal biopsy (or adnexectomy) combined with paclitaxel + platinum perfusion, *G2* peritoneal biopsy (or adnexectomy) combined with pemetrexed + platinum perfusion, *G3* cytoreductive surgery combined with paclitaxel + platinum perfusion, *G4* cytoreductive surgery combined with pemetrexed + platinum perfusion

As shown in Table 3, Ki67 (with > 10% as reference) was not related to survival time (*P>*0.05). Meanwhile, pathological type, T staging, and therapeutic regimen were the key factors affecting the prognosis of MPM patients. Compared with the epithelioid type, the sarcomatoid type had mortality risk increased by 7.663 times (*P* < 0.001). Based on T staging, mortality risk was 2.024 times in stage T_4_ cases compared to stage T_3_ cases (*P=*0.040). Compared to patients administered cytoreductive surgery + pemetrexed + platinum, cases administered peritoneal biopsy (or adnexectomy) + paclitaxel + platinum, peritoneal biopsy (or adnexectomy) + pemetrexed + platinum, and cytoreductive surgery + paclitaxel + platinum had mortality risk increased by 22.794 (*P* < 0.001), 5.797 (*P* < 0.001) and 4.823 (*P =* 0.003) times, respectively; the cytoreductive surgery + pemetrexed + platinum regimen was most effective in prolonging survival time.

## Discussion

The present study showed that prognosis in MPM is associated with pathological subtype, clinical staging, cytoreductive surgery, and subsequent pemetrexed use. In addition, radical cytoreductive surgery and postoperative use of pemetrexed were shown to prolong survival.

MPM may be related to exposure to asbestos [23–25], genetic susceptibility factors, erionite, or SV-40 virus [26, 27]. It has a male predominance, with incidence rates of about 29–58% and 2–23% in males and females upon asbestos exposure, respectively [28]. Of the 52 patients evaluated, 84.6% had 1 to 12 years of asbestos exposure history, and the average age was (60.63 ± 10.32) years.

Treatment methods for MPM at various treatment centers differ. In this study, the median overall survival was 12 months, corroborating previous findings [29]. According to immunohistochemical analysis, there were 46 cases with the epithelioid type and 6 with the sarcomatoid type, indicating a predominance of the former. The MST of patients with the sarcomatoid type was 5 months. Due to the small sample size, statistical bias was high; however, these findings demonstrated to a certain extent that prognosis in the epithelioid type was better than that of the sarcomatoid type, in agreement with previous findings [2]. Multivariate analysis confirmed that the mortality risk of the sarcomatoid type was 7.663 times that of the epithelioid type (*P* < 0.001).

Cox regression model analysis showed that T staging and therapeutic regimen were independent factors affecting patient prognosis in MPM. The PCI scoring system was used to grade the size and involved range of peritoneal tumor tissues, which could better reflect the degree of tumor development. Liang et al. [30] found that the prognosis of patients with stage I-II MPM is significantly better than that of individuals with stage III-IV. In the current study, the 52 patients had PCI scores of 21-39, indicating stage T_3_–T_4_ disease. The mortality risk for stage T_4_ cases was 2.024 times that of patients with stage T_3_ disease (*P =* 0.040). There is currently no consensus or guideline for the treatment of advanced MPM. Treatments include cytoreductive surgery, palliative cytoreductive surgery, hyperthermic intraperitoneal perfusion, and systemic chemotherapy [2]. A retrospective analysis of 4 different therapeutic regimens showed that survival time in patients administered cytoreductive surgery combined with pemetrexed + platinum was the longest, reflecting the best efficacy, with statistically significant differences compared with the other 3 regimens (*χ*^2^ = 30.000, *P* < 0.001). These findings suggest radical cytoreductive surgery as the basis of MPM treatment, which is closely related to overall survival (OS). However, OS differed according to chemotherapy regimen after cytoreductive surgery. Indeed, Cox analysis suggested that the mortality risk of patients administered cytoreductive surgery + paclitaxel + platinum was 4.823 times that of those receiving cytoreductive surgery + pemetrexed + platinum, indicating that the use of pemetrexed could prolong survival. The above results partly contrasted a multi-center study reporting a median OS of 34–92 months and a 5-year survival rate of 59% in patients administered cytoreductive surgery combined with pemetrexed hyperthermic intraperitoneal perfusion [31]. R0 resection is hard to achieve in patients with stage T_3_–T_4_ disease; meanwhile, the complications of radical cytoreductive surgery are relatively abundant, with an incidence of about 27–56% [32]. Such complications include abdominal abscess, anastomotic leakage, severe hypoproteinemia, and lung infection. No serious complications occurred in this study. Some trials may not apply the internationally recommended hyperthermic intraperitoneal perfusion. Therefore, the optimal treatment method is to strictly control surgical indications, reduce complications, completely remove the lesions and combine with subsequent pemetrexed + platinum, therefore prolonging the MST as much as possible.

The Ki67 antigen, one of the most reliable indexes of cell proliferation, can be detected in all active stages of the cell cycle (G1, G2, and S) but is not expressed in the stationary phase. Data collected from 42 MPM patients by Pillai et al. [33] found that low Ki67 is more common in women, with multivariate and univariate analyses showing its positive correlation with prognosis. Deraco et al. [34] analyzed 81 cases, and multivariate prognostic analysis revealed Ki67 >5% as the strongest predictor. Liang et al. [35] analyzed 44 cases of MPM and demonstrated Ki67≧20% is an independent prognostic factor. Li et al. [36] analyzed 25 cases of MPM, and OS in the Ki67 < 20% group was longer than that of individuals with Ki-67 ≥ 20%, although the difference was not statistically significant. In this study, the Kaplan-Meier survival method showed that MSTs in patients with Ki67 ≤ 10% and Ki67 > 10% were 15 and 11 months, respectively. The cumulative survival rates at 12 months were 60.0% and 28.1%, respectively. Therefore, patients with Ki67 ≤ 10% had significantly longer MST and higher cumulative survival rate at 12 months (*χ*^*2*^ = 4.374, *P =* 0.036). In multivariate analysis, Ki67 expression was not an independent factor affecting prolonged survival, which may be related to the limited number of cases in this study.

The limitations of this study should be mentioned. First, this was a single-center study with inherent shortcomings, including a small sample size, the lack of randomization, and the insufficient statistical power. Therefore, large prospective multi-center studies are warranted to confirm these findings. Secondly, clinical data in this study were retrospectively collected, and WT1, BAP1, and p16/CDKN2A, which were previously identified as important prognostic factors in peritoneal mesothelioma, were not detected in patients. In addition, we categorized Ki67 (usually expressed as cardinal numbers), which might induce considerable bias and loss of information due to the underlying great limitation of quantifying immunohistochemical data in a semiquantitative way, although this is usually adopted for pathological parameters.

## Conclusions

In conclusion, T staging and cytoreductive surgery combined with pemetrexed + platinum-based infusion chemotherapy are independent prognostic factors of MPM. Application of pemetrexed could prolong survival time, although long-term survival remains not ideal. Further large-scale multi-center studies are required for the early diagnosis and treatment of MPM.

## Data Availability

The datasets generated and/or analyzed during the current study are not publicly available because we have to carry out a follow-up research, but are available from the corresponding author on reasonable request.

## Abbreviations

CCR: Completeness of cytoreduction
MPM: Malignant peritoneal mesothelioma
MST: Median survival time
MST: Median survival time
OS: Overall survival
PCI: Peritoneal cancer index
